# Peri-implant Soft Tissue Management: Cairo Opinion Consensus Conference

**DOI:** 10.3390/ijerph17072281

**Published:** 2020-03-28

**Authors:** Fernando Suárez López Del Amo, Shan-Huey Yu, Gilberto Sammartino, Anton Sculean, Giovanni Zucchelli, Giulio Rasperini, Pietro Felice, Giorgio Pagni, Vincenzo Iorio-Siciliano, Maria Gabriella Grusovin, Giovanni E. Salvi, Alberto Rebaudi, Giuseppe Luongo, Jack T. Krauser, Martina Stefanini, Andrea Blasi, Jaafar Mouhyi, Faten Ben Amor, Fatme M. Hamasni, Konstantinos Valavanis, Alain Simonpieri, Ahmed M. Osman, Erda Qorri, Rosario Rullo, Arzu Naipoglu, Vincenzo Bruno, Gaetano Marenzi, Francesco Riccitiello, Roberta Gasparro, Nikos Mardas, Gianrico Spagnuolo, Leonzio Fortunato, Hom-Lay Wang

**Affiliations:** 1 Private practice, Tacoma, WA 98465, USA; fsuarezla@gmail.com; 2 Department of Periodontics & Oral Medicine University of Michigan School of Dentistry, Ann Arbor, MI 48109, USA; shanhyu@umich.edu; 3 Department of Neuroscience, Reproductive Science and Dentistry, University of Naples Federico II, 80131 Naples, Italy; enzois@libero.it (V.I.-S.); andreablasi79@gmail.com (A.B.); info@valavanis.net (K.V.); alainsimonpieri@yahoo.com (A.S.); gaetano.marenzi@gmail.com (G.M.); riccitie@unina.it (F.R.); roberta.gasparro88@gmail.com (R.G.);; 4 Department of Periodontology, University of Bern, 3012 Bern, Switzerland; anton.sculean@zmk.unibe.ch (A.S.); giovanni.salvi@zmk.unibe.ch (G.E.S.); 5 Department of Biomedical and Neuromotor Sciences, University of Bologna, 40125 Bologna, Italy; giovanni.zucchelli@unibo.it (G.Z.); pietro.felice@unibo.it (P.F.); martinastefanini3@gmail.com (M.S.); 6 Department of Biomedical Surgical and Dental Sciences, University of Milan, 20122 Milan, Italy; giulio.rasperini@unimi.it (G.R.); giorgio.pagni@gmail.com (G.P.); 7 Vita Salute San Raffaele University, 20132 Milan, Italy; gabri.grusovin@tiscali.it; 8 Private practice, 16100 Genova, Italy; alberto.rebaudi@gmail.com; 9 Private practice, 00100 Rome, Italy; giuseppeluongo@gmail.com; 10 Private practice, U Miami, LSU, USC, U Pittsburgh and Nova Southeastern, Fort Lauderdale, FL 33314, USA; jtkrauser@aol.com; 11 Department of Biomaterial Research, University of Agadir Universiapolis, 80000 Agadir, Morocco; j.mouhyi@hotmail.fr; 12 Department of Anatomy, University of Monastir, 5000 Monastir, Tunisia; faten.benamor@yahoo.fr; 13 Department of Periodontology, Lebanese University, 40016 Hadeth, Lebanon; fatmehamsni@gmail.com; 14 Private practice, 11511 Il Cairo, Egypt; ahmed@osmanhc.com; 15 Faculty of medical Science, Albanian University, 1001 Tirana, Albania; e.qorri@albanianuniversity.edu.al; 16 Multidisciplinary Department of Medical-Surgical and Dental Specialties, University of Campania Luigi Vanvitelli, 80138 Naples, Italy; rosario.rullo@unicampania.it; 17 Department of Head and Neck Surgery, 26480 Istanbul, Turkey; drbnaib@yahoo.com; 18 Department of Biomedical and Speciality Surgical Sciences, University of Ferrara, 44121 Ferrara, Italy; vincbruno@tin.it; 19 The London School of Dentistry & Hospital, London E1 2AD, UK; n.mardas@qmul.ac.uk; 20 Institute of Dentistry, I.M. Sechenov First Moscow State Medical University, 119146 Moscow, Russia; 21 Department of Health Sciences, Magna Graecia University of Catanzaro, 88100 Catanzaro, Italy; leo@unicz.it; 22 Department of Periodontics & Oral Medicine University of Michigan School of Dentistry, Ann Arbor, MI 48109, USA; homlay@umich.edu

**Keywords:** dental implants, peri-implant soft tissue, implant design, keratinized mucosa, soft tissue augmentation

## Abstract

Peri-implant soft tissues play a role of paramount importance, not only on the esthetic appearance, but also on the maintenance and long-term stability of implants. The present report presents the conclusions from the Consensus Conference of the South European North African Middle Eastern Implantology & Modern Dentistry Association (SENAME) (4–6 November 2016, Cairo, Egypt). The conference focused on the topic of the soft tissue around dental implants, and in particular, on the influence of implant configurations on the marginal soft tissues, soft tissue alterations after immediate, early or delayed implant placement and immediate loading, the long-term outcomes of soft tissue stability around dental implants, and soft tissue augmentation around dental implants. Thirty world experts in this field were invited to take part in this two-day event; however, only 29 experts were in the final consensus voting process.

## 1. Introduction

The present report presents the conclusions from the Consensus Conference of the South Europe, North Africa, Middle East Implantology & Modern Dentistry Association (SENAME) (4–6 November 2016, Cairo, Egypt)

Dental implant treatment is currently one of the most common therapies performed in dentistry to restore edentulous ridges. The success of a dental implant treatments not only relies on solid osseointegration between the implant and alveolar bone, but also on soft tissue conditions around the implants, especially when it comes to anterior regions with higher esthetic demands. The SENAME conference was the first worldwide consensus to address peri-implant diseases and conditions. Peri-implant soft- and hard-tissue deficiencies are an independent category to describe the diminished dimensions of the alveolar process/ridge. [1]. Therefore, the experts at this Consensus Conference focused on the topic of soft tissue around dental implants with four main subcategories:

1. Influence of Implant Configurations on Marginal Soft Tissues.

Different implant configuration designs (parallel, divergent, and convergent) are available on the market currently, but limited literature is available regarding how the implant configurations affect the stability of peri-implant soft tissues.

2. Soft Tissue Alterations after Immediate, Early, or Delayed Implant Placement and Immediate Loading.

Dental implant placement has been categorized into four types based on the timing of placement after extraction [2]. It is known that different timing of dental implant placement can influence peri-implant soft tissue, and multiple research projects have investigated their correlation. However, there is no consensus today in terms of how the timing of implant placement could influence soft tissue behavior. Hence, this consensus polled the opinions from world experts concerning their experience in this important topic.

3. Long-term Outcomes of Soft Tissue Stability around Dental Implants.

The success of a dental implant treatment relies not only on osseointegration, but also on the surrounding soft tissue response. For implants placed at maxillary anterior regions with higher esthetic demands, peri-implant soft tissue stability is a critical component for the implant long-term success, as well as patient-reported treatment outcome. Therefore, this conference publicized experts’ opinions about this important topic.

4. Soft Tissue Augmentation around Dental Implants Using Autogenous Soft Tissue Graft or Soft Tissue Graft Substitutes.

Soft tissue augmentation procedures are commonly recommended during implant therapy, in order to gain tissue thickness or keratinized tissue, or to reduce future mucosa recession. To date, opinions in the literature on the materials/techniques that can achieve more predictable and long-term stability remains divided. Therefore, this conference reported the expert opinions on which materials/techniques have the most predictable long-term stability.

Each of the categories was subdivided into different questions responded to by the panel of 29 experts in the field of implant dentistry at the Consensus Conference.

## 2. Influence of Implant Configurations on the Marginal Soft Tissues

### 2.1. Does Implant Design (Parallel, Divergent, or Convergent) Influence Soft Tissue Outcomes?

It was unanimously decided that both parallel and convergent designs lead to better soft tissue outcomes than divergent configurations. However, there is a lack of evidence on this topic, with few studies focusing on different implant designs and their influence on soft tissue outcomes.

### 2.2. Does Implant Platform Switching Design Influence Soft Tissue Outcomes?

When examining the influence of platform switching, divided opinions were obtained, with 52% of members voting yes (*N* = 15) and 48% voting no (*N* = 14). The fact that the results were almost evenly distributed reflects the conflicting views of the research in this area. Platform switching has been widely investigated for its influence on soft and hard tissue changes; however, the impact on the peri-implant soft tissue remains to be further elucidated.

### 2.3. Does Implant Surface Treatment (Smooth vs. Rough Collar) Directly Influence Soft Tissue Outcomes?

Most members of the consensus reported no difference between the implant smooth and rough collar on soft tissue outcomes. Although limited, available evidence corroborates this statement, demonstrating no difference between smooth, moderately rough, and rough surfaces neck implants in terms of soft tissue levels.

### 2.4. Does Implant Surface Treatment (Smooth vs. Rough Collar) Indirectly Influence Soft Tissue Outcomes?

The majority of the members, i.e., 86% (*N* = 25), agreed that surface treatment (e.g., rough collar) on the implant neck area could indirectly influence the soft tissue behavior.

### 2.5. Does Implant Design (Internal vs. External Connection) Influence Soft Tissue Outcomes?

The majority of the members (17 out of 29) agreed that the type of connection does not influence soft tissue outcomes. Although there is very little information on this topic, the available evidence suggests improved outcomes for internal connection on soft tissue levels and papilla fill.

**Figure 1:**
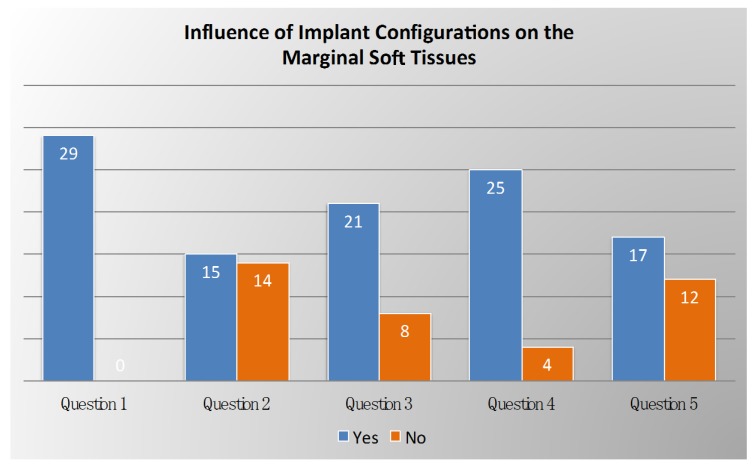
Voting results from the panel of experts regarding the influence of implant configurations on the marginal soft tissues.

Voting results related on the influence of implant configurations on the marginal soft tissues are summarized in Figure 1. 

## 3. Soft Tissues Alterations after Immediate, Early, or Delayed Implant Placement and Immediate Loading

### 3.1. Are There Any Differences in Esthetic Outcomes Between Immediate, Immediate Delayed, and Delayed Implant Placement with Immediate Provisionalization?

The majority of conference members polled (only 14% responded “do not know”) were in agreement about the esthetic outcomes of final soft tissue levels when using different protocols for implant placement. Multiple studies have evaluated the influence of different protocols for implant placement on soft tissue outcomes. Overall, these investigations found differences in soft tissue behavior with the use of different timing for the installation of the fixture.

### 3.2. Are There any Differences on Papillae Fill Between Immediate, Immediate Delayed, and Delayed Implant Placement with Immediate Provisionalization?

Once again, 86% of expert members voted “yes” regarding the influence of these protocols on papilla fill. Investigations have studied the influence of these protocols and reported the different outcomes depending on the timing of provisionalization.

### 3.3. Are there any Differences on Midfacial Mucosal Recession between Immediate, Immediate Delayed, and Delayed Implant Placement with Immediate Provisionalization?

When polled, 72% of members (21 out of 29) agreed that there is less recession of the midfacial gingival level with immediate implant placement and immediate provisionalization. Eight members voted no, saying that more recession occurs after immediate installation and immediate delivery of the prosthesis.

### 3.4. Are There any Differences on Implant Soft Tissue Complications Between Immediate, Immediate Delayed, and Delayed Implant Placement with/without Immediate Provisionalization?

The totality of the panel members voted “yes”, agreeing that there were more soft tissue complications with immediate implant placement with or without immediate provisionalization. It was reported that the immediate installation of the fixture presents with more soft tissue recession, regardless of the loading protocol. Therefore, it is expected that more soft tissue complications will occur. These complications include, but are not limited to, recession defects that may result as a consequence of the immediate installation of the dental implant.

### 3.5. Are There any Differences on Implant Complications (e.g., Peri-implantitis and Implant Failure) Between Immediate, Immediate Delayed, and Delayed Implant Placement with/without Immediate Provisionalization?

Conference members unanimously agreed that there were more complications with immediate implant placement with or without the immediate installation of the provisional restoration. As such, the immediate installation of the fixture after extraction represents a more challenging and technique-sensitive procedure than delayed implant placement. Furthermore, seeking primary stability may compromise the positioning of the implant in immediate scenarios, resulting in less than ideal positions.

**Figure 2:**
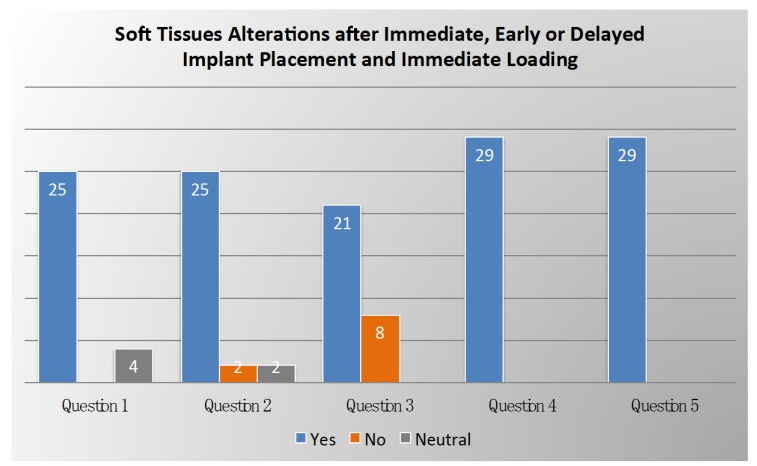
Voting results from the panel of experts on soft tissue alternations after immediate, early, or delayed implant placement and immediate loading.

Voting results on soft tissue alterations after immediate, early or delayed implant placement and immediate loading are summarized in Figure 2.

## 4. What Is the Long-Term Stability of Keratinized Mucosa at Osseointegrated Implants? 

### 4.1. Does More than 2 mm of Attached/Keratinized Mucosa width Influence Long-Term (e.g., >5-Year) Peri-implant Soft Tissue Stability (e.g., Midfacial Mucosal Recession)?

The presence of more than 2 mm of attached/keratinized mucosa was reported to influence the long-term peri-implant soft tissue stability by 25 members, while four panel members reported they were neutral about this specific topic. The influence of a certain amount of attached/keratinized mucosa around dental implants has been a topic of major interest over the years. Multiple studies have demonstrated the beneficial effect of this band of attached/keratinized tissue, especially in the rough surface implants in several aspects, including plaque accumulation, attachment loss, inflammation and/or infection, and recession.

### 4.2. Does a Mucosa Thickness of 2 mm or more Influence Long-Term (e.g., >5-Year) Peri-implant Soft Tissue Stability (e.g., Midfacial Mucosal Recession)?

The presence of a mucosa with a thickness of 2 mm or more was determined to influence long-term peri-implant soft tissue stability by all but one panel member, who reported being neutral about this topic. Although not widely investigated, the beneficial effect of this has been studied since the 1990s. As such, it has been demonstrated that a certain amount of mucosal thickness is necessary for the establishment of the biological width around the fixture. If thin tissue is present, crestal bone resorption will occur, providing more space for both junctional epithelium and connective tissue formation. Consequently, although there is a lack of evidence concerning implant soft tissue stability and its relation to tissue thickness, it is possible to assume that the maintenance of crestal bone levels will ultimately dictate the maintenance of midfacial mucosal levels.

### 4.3. Does the Soft Tissue Mobility Influence Long-Term (e.g., >5-Year) Peri-implant Soft Tissue Stability (e.g., Midfacial Mucosal Recession)?

Complete agreement was obtained concerning the notion that the presence of movable peri-implant tissue can alter the long-term stability of the midfacial mucosal gingival margin. Although this aspect has been poorly described in the literature, it may be possible that plaque accumulation and inflammation occur in most instances and to a greater extent over unattached or movable tissue. The presence of movable tissue can be related to the lack of attached and/or keratinized tissue. 

**Figure 3:**
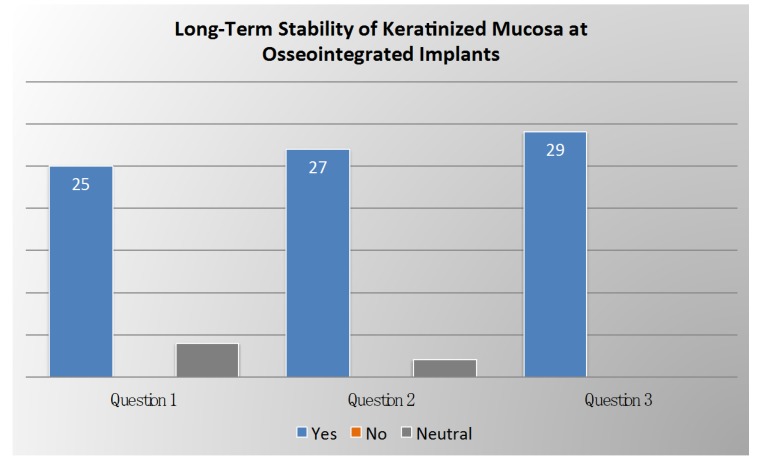
Voting results from the panel of experts regarding the long-term stability of keratinized mucosa at osseointegrated implants.

Voting results on long-term stability of keratinized mucosa are summarized in Figure 3. 

## 5. The Effectiveness of Attached and Keratinized Tissue Augmentation around Dental Implants: Autogenous Versus Soft Tissue Substitutes

### Do Autogenous Soft Tissue Grafts Achieve Better, Similar, or Worse Keratinized Tissue Augmentation around Dental Implants When Compared to Soft Tissue Substitutes Grafts (e.g., Acellular Dermal Matrix or Collagen Derivatives)?

When comparing the results of keratinized and attached mucosa augmentation using autogenous soft tissue grafts versus soft tissue substitutes/replacement grafts, all members supported the superiority of autogenous grafts. A multitude of studies and systematic reviews in the field corroborate this statement, demonstrating the superiority of connective tissue grafts for multiple parameters for attached/keratinized mucosa augmentation.

**Figure 4:**
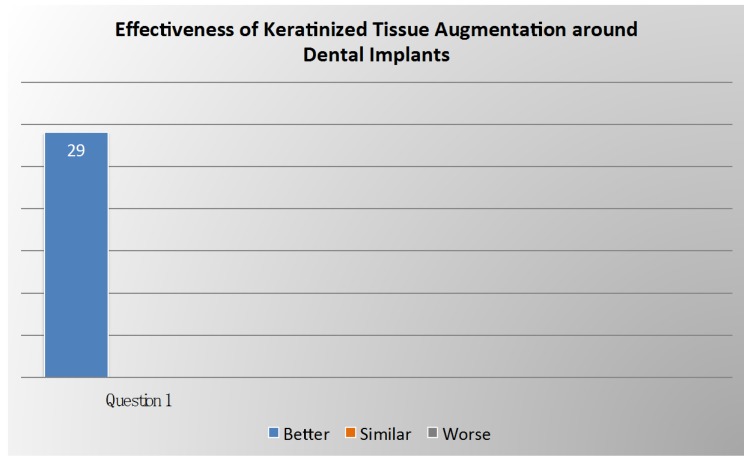
Voting results from the panel of experts regarding the effectiveness of keratinized tissue augmentation around dental implants.

Voting results related on effectiveness of keratinized tissue augmentation around dental implants are summarized in Figure 4. 

## 6. Discussion

The peri-implant soft tissues play a role of paramount importance, not only on the esthetic appearance, but also on the maintenance and long-term stability of fixtures. Fixture outcome has been evaluated depending on several influential factors, including but not limited to the presence of keratinized and attached mucosa [3], the tissue thickness [4], the different loading protocols [5], and soft tissue grafting techniques [6], among others. While the beneficial effect of a band of attached and keratinized tissue has been widely reported [7], several other parameters require further investigation. Moreover, scientific evidence has reported mixed results. The present review aimed to report the expert opinions of those in the field of peri-implant soft tissues and their clinical experience on daily cases to further evaluate the different aspects of peri-implant soft tissues and the modifying factors that may play a role therein. Four main categories were evaluated, namely: the influence of implant configurations, implant placement and loading protocols, soft tissue augmentation procedures, and the stability of the same.

The influence of the implant characteristics and the implant-abutment interface have been more extensively studied with regard to bone remodeling and marginal bone loss [8,9]. Although directly influenced by the underlying bone morphology, a comprehensive understanding of the influence of these parameters on the implant marginal soft tissues is lacking. Factors such as the type of connection (i.e., internal vs. external) as well as the utilization of platform switching resulted in divided opinions among the panel of experts. On the other hand, the majority of the voting members agreed that the surface treatment to the implant neck only plays an indirect role on soft tissue outcomes. Finally, panel members were in full agreement that parallel and convergent implant designs produced superior outcomes compared to divergent configurations.

The use of different types of protocols for implant installation and subsequent loading have been widely investigated. It was unanimously agreed that immediate implant placements result in more complications than delayed implant placements. Available evidence corroborates the presence of higher soft tissue complications and implant failures with immediate implant placements [10]. Immediate implant placements and provisionalization produced less midfacial recession, according to panel members. Nevertheless, most of the members reported that they were unsure about the influence of implant installation protocol on esthetic outcomes. These results seem to be in concordance with recent investigations demonstrating exceptional results after immediate implant placement and provisionalization [11,12,13].

The influence of keratinized and attached mucosa was also evaluated at this conference. Panel members agreed that autogenous tissue replacement grafts were superior to soft tissue replacement grafts for augmentation procedures around dental implants. Similarly, the majority of members agreed on the importance of more than 2 mm of attached/keratinized mucosa and a mucosa thickness of 2 mm or more for long-term peri-implant soft tissue stability. It was also agreed that the presence of movable peri-implant tissue can alter the long-term stability of the gingival margin. The results obtained in this consensus concerning the importance of the keratinized/attached tissue and the thickness of the same are in concordance with previous investigations [3].

## 7. Conclusions

Soft tissue conditions around the implants must be always evaluated, especially when it comes to anterior regions with higher esthetic demands. The SENAME conference was the first consensus to discuss peri-implant diseases and soft tissue conditions among worldwide experts.
